# The Diet of To-Day

**Published:** 1907-01-19

**Authors:** W. D. Halliburton

**Affiliations:** Professor of Physiology, King's College, London


### THE DIET OF TO-DAY.

By W. D. HALLIBURTON, M.D., F.R.C.P., F.R.S., Professor of Physiology, King's College, London.

(*Specially reported for* THE HOSPITAL.)

IN discussing the question of the most suitable diet for the healthy, Professor Halliburton, at the Incorporated Institute of Hygiene on Wednesday, January 9, delivered a lecture incorporating certain features of the problem. Diet constituents appear to be of an extremely diverse character, but when examined it is found that the number of substances is after all not very numerous. There are inorganic foods, those derived from the mineral world, and water, oxygen of the air, and the various salts, but principal importance attaches to the derivatives of the organic kingdom, substances which have life, either vegetable or animal. The first of the three main kinds are proteids or albuminous substances, found in eggs, meat, or milk. The second class are carbohydrates, substances of the nature of starch and the various forms of sugar; and the third including substances which are called fats—compounds of glycerine and various fatty acids. These contain carbon, hydrogen, and oxygen in certain proportions, and the proteids contain in addition to these the important element nitrogen, answering for the building up of the protoplasm of the body and are spoken of as nitrogenous foods—the fats and carbohydrates as non-nitrogenous. Professor Voigt, of Munich, formulates the proportion in which these substances occur in the standard diet, putting the proteids required at 100 grammes, equivalent to ¼ lb. If all proteids were taken in the form of meat, 1 lb. of meat would be required in the 24 hours. Milk contains 10 per cent., and cheese is pure proteid. Starch and sugar form the main constituents of our diet and furnish about 250 grammes, and the fats about 100. Baron Liebeg grouped the dietary in another way. The proteids or nitrogenous he regarded as flesh-forming. The other forms of food he denominated heat or energy producers. This classification does not recognise the fact that proteids are also a source of energy. Within the last few years Professor Chittenden, of Yale, has devoted much enthusiasm to the investigation of this subject and has made some experiments of first-class importance by placing numbers of persons on a rigid regimen and reducing the 100 grammes of proteid down to 50 grammes. Chittenden is a strong advocate for temperance in the eating of meat and other forms of proteid food, and concludes that the amount of albuminous food indulged in by the greater number of meat-eating nations is a good deal too high. While sympathising with Professor Chittenden for his advocacy of temperance, Dr. Halliburton thought the adoption of his views was fraught with danger.

Professor Halliburton pointed out that while nitrogenous food is essential for the repair of tissue waste, the terms nitrogenous and nutritious must not be regarded as synonymous. Non-nitrogenous foods are equally necessary for the preservation of good health, as these supply energy and animal heat. Undoubtedly, a large amount of proteid food is not required for tissue repair, which means that its excretion causes waste of energy. It is, therefore, regrettable that most well-to-do persons eat more meat and drink more alcohol than is good for their health. Nevertheless, Professor Halliburton did not think it wise to accept Professor Chittenden's views in full, for the reason that the minimum diet is not necessarily the optimum. In support of this argument the lecturer gave as examples the dietary of the poor in large cities, and the small amount of nitrogenous matter eaten by vegetarians. Both classes, he considered, showed less resisting power to hardship and disease than those who were meat eaters. The beneficial effects of generous feeding in pulmonary tuberculosis, and of the rest cure and good feeding for various nervous diseases were here quoted in support of the lecturer's attitude. He was, he said, of the opinion that the amount of proteid food should not be limited to subserve the repair of waste tissue, for this means to live on the margin of danger; but he thought that experiments on a far wider scale than Professor Chittenden's must be carried out before dogmatic statements would be safe.

Physiologists had called attention to many considerations in connection with Chittenden's work which made one pause before accepting his conclusions. It had been calculated by Dr. Loathes that the amount of nutriment provided to the infant by the mother's milk was about ten times that of Chittenden's minimum, and even allowing for the growth of the infant, what is true of one period of life is probably true in others. It was not considered physiologically necessary to take food which would yield the least amount of refuse either nitrogenous or otherwise. In this connection experiments on animals are not nearly so convincing as experiments made upon man. For if the vegetarian can point to the large amount of work done by animals living upon a very dilute proteid nutriment, the meat eater can point to the lion, in which exactly the opposite is true. The poor feed on a diet which is very like Chittenden's, and their nutritive condition is not such as to induce others, who can afford a more liberal diet, to follow their example. In Oriental countries the meat eaters have come to the forefront and can withstand disease, fatigue, and privation most easily. In reference to the Japanese the lecturer pointed out how sadly needed are accurate details of what this nation exactly does consume. There is no doubt that within recent years their vegetarian habits have been largely departed from, and some writers have, in part, attributed their recent vigour and success to this very circumstance. Referring to the Japanese, who are always adduced as models of plain living combined with efficiency, Professor Halliburton drew attention to the fact that no accurate information had been forthcoming as to whether the diet of our allies was so very poor in proteids as had been alleged. The supposition that the Japanese subsisted wholly on vegetable products could not be supported. Undoubtedly, in recent years, their vegetarian habits had been largely departed from, so that it was open to ask whether their recent vigour and success may not have had an exactly opposite origin to that so often attributed. In resistance to disease there was nothing like a good appetising diet. The open-air cure for consumption has been spoken of as the over-feeding cure, and in the rest-cure treatment for nervous troubles, there is no doubt that, abundant food materials enabled the body the better to resist the disease. To maintain in efficiency the phagocytes it seemed to be necessary to have a very large excess of nutrient material in the blood which surrounds them. The real explanation of the usefulness of an apparent excess is that certain constituents of the proteid molecule are particularly essential for the building up of the tissues. The amount of these necessary constituents is very limited in the proteid molecule, and in order to obtain an adequate supply the body has to put up with a good deal of waste substance, but the large size of the liver appears to be an express provision of nature for dealing with this waste rapidly. The easy digestibility of the proteids of animal origin render them superior to those from the vegetable world, and the so-called vegetarian has had perforce to recognise this by adding to his diet three of the richest products from the animal kingdom—namely, milk, cheese, and eggs.

Speaking of milk, Professor Halliburton said it had been called a perfect food because it contains all the different kinds of compounds in suitable proportions, and should be used as the exclusive diet of infancy, but it was like the voice of one crying in the wilderness to preach that doctrine to the public at large. They are proud of the fact that their babies will "eat anything." The milk supply should be pure, and this ought to form one of the front-rank subjects for the attention of the Government. Preservatives often concealed the presence of germs of disease and acted harmfully upon digestion and upon the digestive ferments. In legislation for the provision of an adequate milk supply for infants and invalids the prohibition of preservatives should form a factor.

Diet was the subject of a great many fads. The most dangerous talkers were those with a little knowledge. Thus Mr. Horace Fletcher was the apostle of thorough mastication, but no amount of chewing could put more energy into food than it already had. The law of the conservation of energy was one of the truest in physiology. To suppose that by the act of thorough mastication one could make food more valuable than it was previously was nonsense. The modern tendency is towards moderation in both eating and drinking, though it is improbable that the "tablet" diet is ever likely to be introduced into public favour. There is the æsthetic and psychical element to be considered, and that is particularly true of those with weak digestion, and of invalids. The humanitarian and sentimental argument used by the vegetarians has received powerful support from the recent disclosures. But vegetarianism, if carried too far, may produce greater horrors even than the Chicago stockyards. For well-meaning but ignorant people had already carried vegetarianism to excess and imposed a great deal of unnecessary suffering upon children. It is alleged that a flesh diet is bad for gout. No doubt it is the case that certain extractives in meat are particularly harmful in gout and allied disorders. One cannot prescribe as a proper diet for the healthy one which is necessary for gouty sufferers. Certain forms of disease require certain forms of diet. For instance, one cannot advise vegetarians to abstain from carbohydrate food because that form of diet is bad for diabetics.

